# Plasmin drives burn-induced systemic inflammatory response syndrome

**DOI:** 10.1172/jci.insight.154439

**Published:** 2021-12-08

**Authors:** Breanne H. Y. Gibson, Colby C. Wollenman, Stephanie N. Moore-Lotridge, Patrick R. Keller, J. Blair Summitt, Alexey R. Revenko, Matthew J. Flick, Timothy S. Blackwell, Jonathan G. Schoenecker

**Affiliations:** 1 Department of Pharmacology;; 2 School of Medicine;; 3 Department of Orthopaedic Surgery, Vanderbilt University Medical Center;; 4 Vanderbilt Center for Bone Biology;; 5 Department of Plastic Surgery, Vanderbilt University Medical Center; and; 6 Vanderbilt University Medical Center Burn Center, Vanderbilt University, Nashville, Tennessee, USA.; 7 IONIS Pharmaceuticals Pulmonary and Oncology Drug Discovery, Carlsbad, California, USA.; 8 Department of Pathology, University of North Carolina-Chapel Hill, Chapel Hill, North Carolina, USA.; 9 University of North Carolina Blood Research Center, Chapel Hill, North Carolina, USA.; 10 Department of Cancer Biology, Vanderbilt University, Nashville, Tennessee, USA.; 11 Division of Pulmonary and Critical Care;; 12 Department of Pathology, Microbiology, and Immunology; and; 13 Department of Pediatrics, Vanderbilt University Medical Center, Nashville, Tennessee, USA.

**Keywords:** Inflammation, Cytokines, NF-kappaB, Plasmin

## Abstract

Severe injuries, such as burns, provoke a systemic inflammatory response syndrome (SIRS) that imposes pathology on all organs. Simultaneously, severe injury also elicits activation of the fibrinolytic protease plasmin. While the principal adverse outcome of plasmin activation in severe injury is compromised hemostasis, plasmin also possesses proinflammatory properties. We hypothesized that, following a severe injury, early activation of plasmin drives SIRS. Plasmin activation was measured and related to injury severity, SIRS, coagulopathy, and outcomes prospectively in burn patients who are not at risk of hemorrhage. Patients exhibited early, significant activation of plasmin that correlated with burn severity, cytokines, coagulopathy, and death. Burn with a concomitant, remote muscle injury was employed in mice to determine the role of plasmin in the cytokine storm and inflammatory cascades in injured tissue distant from the burn injury. Genetic and pharmacologic inhibition of plasmin reduced the burn-induced cytokine storm and inflammatory signaling in injured tissue. These findings demonstrate (a) that severe injury–induced plasmin activation is a key pathologic component of the SIRS-driven cytokine storm and SIRS-activated inflammatory cascades in tissues distant from the inciting injury and (b) that targeted inhibition of plasmin activation may be effective for limiting both hemorrhage and tissue-damaging inflammation following injury.

## Introduction

The acute phase response (APR) is the physiologic process by which the body survives and resolves tissue injury. Following a nonsevere injury, the APR is comprised of initial, synergistic activation of coagulation and inflammation to contain the damage and prevent infection at the site of injury. Once adequate containment is achieved, repair mechanisms are activated to remove the damaged tissue and promote regeneration to restore tissue function (1). A severe injury provokes a deranged APR that imposes global, pathologic effects on distant organ systems. Consequently, severe injuries are leading sources of morbidity and mortality due to critical adverse outcomes (2). The hallmark of a pathologic, severe injury–induced APR is dysregulated inflammation and coagulation (3, 4). The inflammatory pathology is referred to as systemic inflammatory response syndrome (SIRS) highlighted by a “cytokine storm” (5). The coagulopathy is referred to as a trauma-induced coagulopathy (TIC), which includes coincident paradoxical bleeding and thrombotic phenotypes (6, 7). Together, SIRS and TIC result in an “immunocoagulopathic” condition that affects the vasculature and peripheral tissues, increasing the risk of multiple-organ dysfunction syndrome (MODS; refs. 4, 8, 9) and impeding tissue repair later in convalescence (10–12). A critical knowledge gap is apparent regarding molecular mechanisms that initiate this detrimental immunocoagulopathy in a severe injury–induced APR.

In the context of a nonsevere injury APR, the protease plasmin is activated locally at the site of the damage within days following injury, where it removes fibrin (fibrinolysis) and other matrices and activates growth factors, which together stimulate tissue regeneration (1). While plasmin’s conventional role is intravascular and extravascular fibrinolysis, plasmin also modulates inflammation through a plethora of mechanisms, including cellular signaling, matrix removal, and activation of cytokines and other proteases (13–16). In the context of a maladaptive APR following a severe injury, excess, early, systemic activation of plasmin has been shown to perpetuate lethal clinical outcomes. The most characterized adverse outcome of systemic pathologic plasmin activation following severe injury is blood loss due to inappropriate clot degradation (hyperfibrinolysis; refs. 17, 18). Clinical trials investigating the use of antifibrinolytic drugs have clearly demonstrated that timely prevention of plasmin activation reduces both blood loss, transfusion, and death (19, 20). In addition, these studies also demonstrate reduction in markers of inflammation, but it is unclear if the antiinflammatory effects of antifibrinolytics are secondary to a reduction in bleeding (21). These data suggest a context-dependent contribution for plasmin activation following severe injury, exacerbating inflammation in severe injury but functioning to resolve inflammation following mild injuries. In disease states, such as cancer, arthritis, and infection, plasmin has been identified as a pathologic instigator of inflammation (22–24), but its isolated role in severe injury–induced SIRS, including the cytokine storm and activation of inflammatory pathways within injured tissue, is unknown.

In this study, we investigated the role of plasmin in the deranged inflammatory component of a pathologic APR, assessing its effects on cytokine storm and tissue-specific inflammatory signaling. We hypothesized that severe injury–induced excess plasmin activity is a key molecular driver of SIRS. A major barrier to investigating the relationship between injury-induced plasmin activity and inflammation is the presence of bleeding because of hyperfibrinolysis, which is often fatal and may instigate a secondary inflammatory response. Following severe injury, deaths can occur in a trimodal distribution: immediate, early due to hemorrhage, and late due to SIRS-associated MODS or infection (25, 26). In many preclinical models of severe injury, early mortality prevents the study of the later causes of mortality. To circumvent bleeding as both a confounding variable and a significant cause of early death after severe injury, we investigated plasmin activation in both clinical and preclinical models of severe burn in which SIRS occurs without the risk of lethal blood loss.

## Results

### Clinical cohort description.

As a part of a prospective study, 31 patients with partial and full-thickness cutaneous burns affecting 5%–95% of their total body surface area (TBSA) and 15 healthy control subjects were enrolled following informed consent (Table 1) for blood collection. Flash burns were the most common mechanism of injury (77%, 24 of 31), with 42% (13 of 31) of patients experiencing concomitant inhalation injury (Table 2). Patients experiencing burn injury were found to have a median revised Baux score of 79 (range 9–119) at the time of admission, with in an in-hospital mortality rate of 19% (6 of 31; Table 2). Throughout hospitalization, 71% of patients (22 of 31) required surgical intervention for treatment of their burn injuries. Within the first week of hospitalization, 87% (27 of 31) of burn patients developed markers of organ dysfunction based on the Sequential Organ Failure Assessment (SOFA) scoring system (27), and 42% (13 of 31) developed multiple organ dysfunction defined as a SOFA score ≥ 6 (Table 2).

### Burn-induced SIRS and coagulopathy.

**Figure 1:**
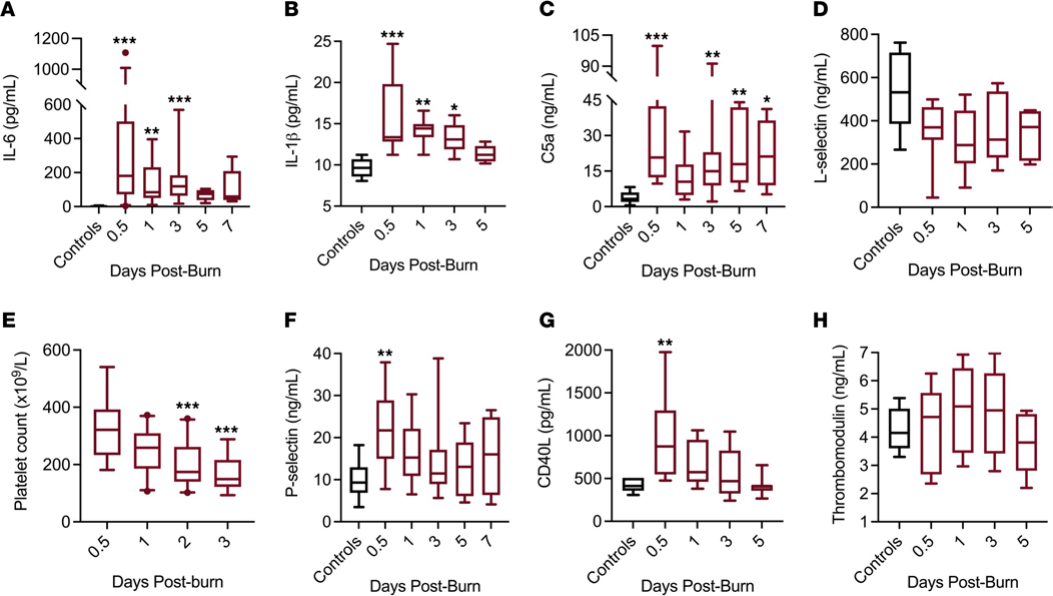
SIRS and coagulopathy in burn patients. (**A**–**D**) Burn patients exhibit markers of inflammation, including (**A** and **B**) significantly elevated inflammatory cytokines (IL-6, IL-1β) and (**C**) markers of complement activation (C5a) upon admission, and (**D**) a reduction in leukocyte activation marker soluble L-selectin throughout hospitalization. (**E**–**H**) Burn patients exhibit hallmarks of coagulopathy including (**E**) decreased platelet count with high levels of platelet activation markers, (**F**) P-selectin, (**G**) CD40L, and (**H**) endothelial activation marker thrombomodulin. (*P < 0.05, **P < 0.01, ***P < 0.001 compared with controls. A Kruskal-Wallis with Dunn’s post hoc correction for multiple comparisons was performed; *n =* 24–31 for 0.5, 1, 3, days, and *n* = 7–15 for 5 or 7 days due to mortality or discharge; *n =* 15 for controls. Boxes represent median, while whiskers represent the 5th–95th percentile.

Severe burns are unique injuries that provoke SIRS and coagulopathy without a significant risk of bleeding prior to surgical intervention (28, 29). In this cohort, 87% (27 of 31) of patients developed SIRS within the first 72 hours of the burn based on clinical criteria, including dramatic changes in heart and respiratory rate, temperature, and white blood cell count (ref. 30 and Table 2). In addition to clinical criteria, the burn patients we analyzed exhibited other indications of SIRS, including high plasma concentrations of inflammatory cytokines IL-6 and IL-1β (refs. 5, 31; Figure 1, A and B), consistent with previous findings in burn patients (32, 33). Another hallmark of systemic inflammation closely linked with coagulation activation and plasmin activation is complement activation (16, 34, 35). In our cohort, burn patients exhibited high levels of C5a, indicative of complement activation, at the time of admission. Throughout hospitalization, C5a levels fluctuated relative to surgical interventions, which represent additional instances of tissue injury and introduce the risk of bleeding (Figure 1C). Interestingly, soluble L-selectin, a common marker for leukocyte activation, decreased, although not significantly, during hospitalization (Figure 1D), consistent with previous findings in trauma patients (36).

In addition to changes in SIRS, burn patients also exhibit hallmarks of coagulopathy, including decreased platelet count, excess thrombin generation, and markers of platelet activation due to injury severity and high-volume crystalloid fluid resuscitation (29, 37, 38). Upon admission and prior to surgical intervention, burn patients exhibited signs of coagulation activation marked by significantly decreased platelet counts within 1.5 days after burn, with the average platelet count nadir occurring at 2 days after burn, along with elevated soluble P-selectin and CD40L in the plasma, indicative of platelet activation (Figure 1, E–G). Previous studies have demonstrated increased circulating thrombomodulin as an indicator of endothelial dysfunction in patients with severe burns (37), but in this heterogeneous cohort, we observed only a minor, nonstatistically significant increase in plasma thrombomodulin on average (Figure 1H).

### Plasmin activation and fibrinolysis with reduced circulating plasminogen levels in burn patients.

**Figure 2:**
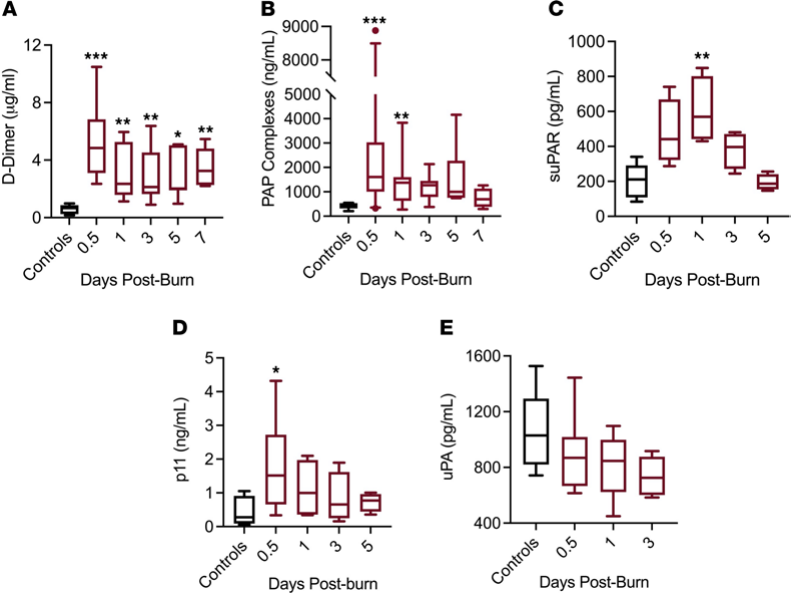
Plasmin activation and fibrinolysis in burn patients. (**A** and **B**) Burn patients exhibit significant (**A**) plasmin activation and (**B**) fibrinolysis within the first day of the burn. (**C**) Soluble uPAR was elevated within the first 3 days of the burn. (**D**) Soluble endothelial p11 was elevated upon admission prior to approaching baseline around 3 days post-burn. (**E**) Free uPA levels decreased throughout hospitalization. Kruskal-Wallis with Dunn’s post hoc correction for multiple comparisons was performed. *P < 0.05, **P < 0.01, ***P < 0.001 compared with controls. For **A** and **B**: *n =* 24–31 for 0.5, 1, 3, days, and *n* = 7–15 for 5 or 7 days; for **C** and **D**, *n =* 15 for controls. Boxes represent median, while whiskers represent the 5th–95th percentile.

Current data on plasmin activation in burn are controversial with some studies that suggest patients do not exhibit hyperactivation of plasmin, as measured by clot lysis on thrombelastography (TEG; ref. 39), while other studies employing alternative measurements of plasmin activity suggest significant plasmin activation immediately following burn (40). In this study, we found that burn patients exhibited significant activation of plasmin activation. D-dimer levels were significantly elevated in all patients upon admission and normalized within 1–2 days of admission if no surgical intervention was initiated (Figure 2A). Furthermore, the burn patients in this study were found to exhibit circulating plasmin-antiplasmin (PAP) levels 10- to 50-fold greater than normal values within the first 12 hours following burn injury (Figure 2B). Therefore, burn does provoke significant, early activation of plasmin and fibrinolysis.

Furthermore, levels of proteins previously suggested to play a role in aberrant plasmin activation following trauma — circulating soluble urokinase plasminogen (Plg) activator (uPA), uPA receptor (suPAR), and soluble endothelial protein p11 (S100A10) (41, 42) — were significantly elevated within the first 1–3 days after burn (Figure 2, C and D). However, detection of free uPA decreased throughout hospitalization following rapid plasmin activation (Figure 2E).

**Figure 3:**
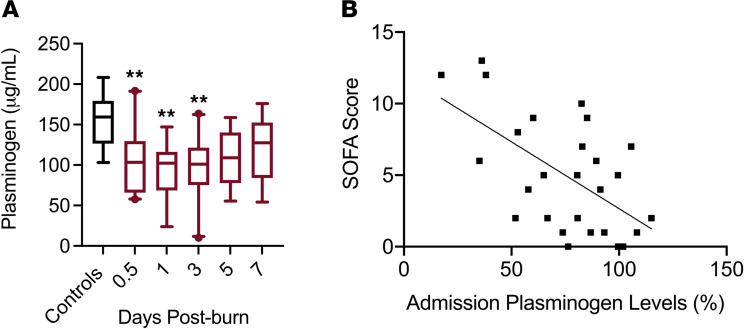
Decreased Plg levels in burn patients are associated with organ failure. (**A**) Plg levels in burn patients were below normal values upon admission and remained low throughout hospitalization. Kruskal-Wallis with Dunn’s post hoc correction. **P < 0.01 compared with controls; *n =* 24–31 for 0.5, 1, 3, days, and *n =* 15 for 5 or 7 days, *n =* 15 for controls. Boxes represent median, while whiskers represent the 5th–95th percentile. (**B**) Admission plasma Plg levels were inversely correlated with the magnitude of organ failure incurred within the first week of hospitalization (Pearson correlation, *R*^2^ = 0.343, *P =* 0.0013).

All patients with burns presented with decreased circulating levels of Plg, and those with severe burns (>20% TBSA, *n =* 20 of 31) exhibited 50%–75% decreases in Plg (Figure 3A). Upon admission, circulating Plg levels were inversely associated with the percent TBSA affected by the burn (*R*^2^ = 0.487, *P* ≤ 0.0001). Across the first 7 days following burn injury, Plg levels remained 40%–60% of normal levels. In addition to redistribution, other studies have demonstrated that significant plasmin activation can result in lower circulating Plg levels (40). The significant increase in PAP (Figure 2B) suggests that at least a portion of the observed decrease in Plg is due to plasmin activation and subsequent clearance from the blood. While the clinical significance of diminished Plg is currently unknown, this study found that Plg depletion from the plasma at the time of admission strongly correlated with the development of organ dysfunction in the first week of hospitalization (*R*^2^ = 0.343, *P =* 0.0013; Figure 3B).

### Plasmin in burn-induced SIRS.

**Figure 4:**
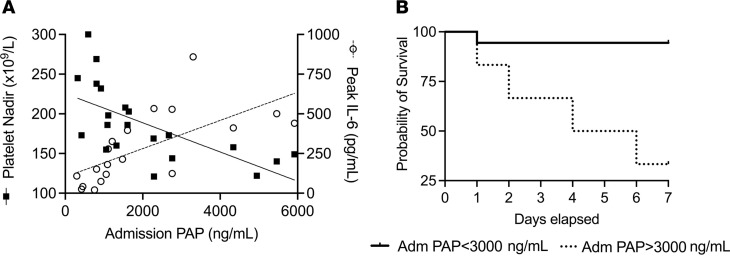
The magnitude of early plasmin activation is associated with markers of SIRS, coagulopathy, and in-hospital mortality. (**A**) Plasmin activation within 12 hours of the burn correlated with patient platelet nadir prior to surgical intervention (*R*^2^ = 0.425, *P =* 0.0014) and peak plasma IL-6 (*R*^2^ = 0.445, *P =* 0.0018) by Pearson correlation. (**B**) Admission PAP levels > 3000 ng/mL were significantly associated with 7-day mortality (log-rank test, *P =* 0.0012, HR = 14.73). Patients at risk: PAP *<* 3000 ng/mL (*n* = 18), PAP > 3000 ng/mL (*n* = 6); censored subjects (patients alive at 7 days) (*n* = 17 and 2), respectively. *n =* 24 patients admitted within 12 hours of the burn.

Previously, the significance of early, exuberant plasmin activity in severe injuries without bleeding was unknown. In this study, early PAP values positively correlated with peak plasma IL-6 values (*R*^2^ = 0.445, *P =* 0.0018) and negatively correlated with the lowest platelet count in patients prior to surgical intervention (*R*^2^ = 0.425, *P =* 0.0014), thereby associating with both inflammation and coagulopathy markers, respectively (Figure 4A). Furthermore, admission PAP values > 3000 ng/mL were found to associate with mortality within 7 days of burn injury (*P =* 0.0012, HR = 14.73; Figure 4B). However, these correlative findings do not confirm a causative role for plasmin in burn-induced inflammation and may represent an association between factors within the severe APR. Therefore, we implemented murine models to investigate the mechanistic relationship between early plasmin activation and burn-induced SIRS.

### Mouse model of burn-induced SIRS.

**Figure 5:**
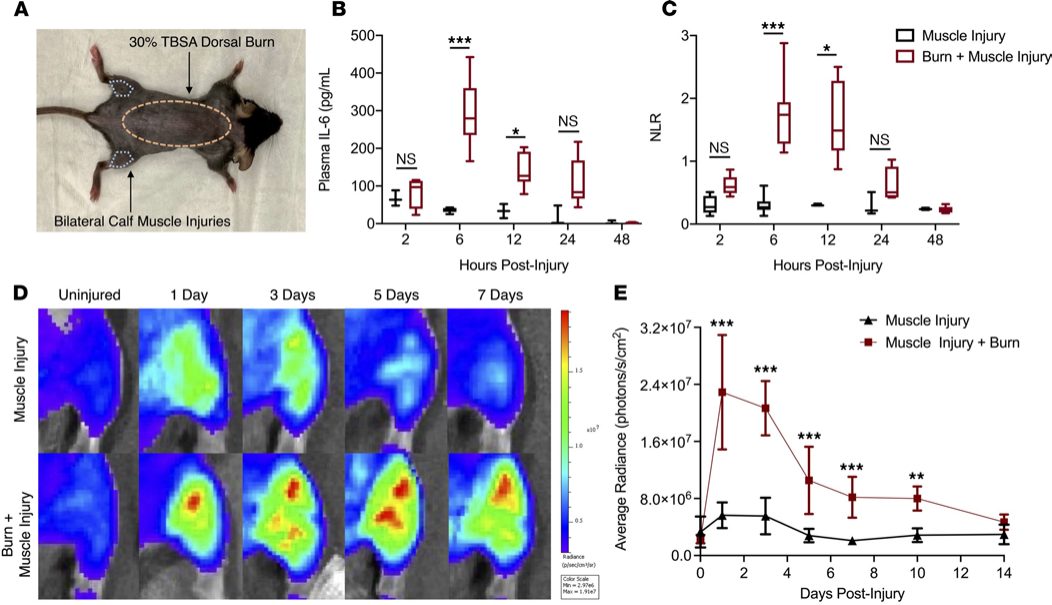
A mouse model of burn polytrauma provokes a systemic inflammatory response, affecting circulating and tissue markers of inflammation. (**A**) Mouse model of 30% TBSA dorsal burn injury with concomitant, bilateral calf muscle injuries. (**B** and **C**) Plasma IL-6 and neutrophil to lymphocyte ratio (NLR) were significantly increased within 6 to 12 hours of the burn injury compared with the muscle injury alone, which does not significantly increase either marker. (**D** and **E**) NF-κB signaling in an isolated calf muscle injury is increased 1 to 3 days after injury and is resolved by 7 days. *n =* 5/group. Boxes represent median, while whiskers represent the 5th–95th percentile; multiple Mann-Whitney *U* tests at each time point with Holms-Sidak correction for multiple comparisons. *P < 0.05, ***P < 0.001, compared with muscle injury alone. The presence of a dorsal burn distant from the site of muscle injury increases and augments NF-κB signaling in injured muscle. Repeated measures ANOVA, **P < 0.01, ***P < 0.001 compared with muscle injury alone. *n =* 7 mice/group; points represent mean ± SD with 95% CI.

To investigate the mechanistic role of plasmin in burn-induced SIRS, we used a well-established murine severe burn model, in which SIRS is known to occur (43). Although SIRS is typically measured by circulating markers and basic vital signs, the inflammation provoked by severe injuries is systemic, affecting distant organs and other injured tissues. Many severe burn injuries are not isolated to a dermal wound alone, but also involve concurrent injuries to muscle, bone, and nerves (44). Therefore, we used a validated model of burn with concomitant muscle injuries remote from the site of burn (45). To provoke a nonsevere APR, isolated calf muscle injuries were administered by cardiotoxin injection. In contrast, to provoke a severe APR, a 30% TBSA dorsal burn was conducted in combination with the same isolated calf muscle injuries (Figure 5A). On a cytokine panel, we observed the greatest difference in plasma between muscle injury alone and burn with muscle injury in IL-6 measurements (Supplemental Figure 1; supplemental material available online with this article; https://doi.org/10.1172/jci.insight.154439DS1), and this difference in plasma has been shown to be an accurate predictor of SIRS and MODS (5, 31, 46). Therefore, to measure systemic markers of SIRS in the blood, we quantified plasma IL-6 and neutrophil/lymphocyte ratio (NLR) as markers of inflammation and innate immune response. Aligning with a more severe APR, mice with the burn injury developed a significant increase in circulating IL-6 and NLR that peaked within 6 hours after injury and resolved within 24–48 hours compared with those that received muscle injuries alone (Figure 5, B and C).

### Burn injury augments and prolongs NF-κB signaling at a site of injury remote to the burn.

To measure the effects of burn on tissue-specific inflammation at sites of injury remote to the site of burn, we implemented the burn model with concurrent, anatomically remote, bilateral muscle injuries (as described above) in mice expressing an NF-κB–inducible luciferase reporter (47). Transcription factor NF-κB, a central mediator of many convergent cellular inflammatory pathways, is activated within the muscle immediately following injury and is resolved within 5 days following injury (Figure 5, D and E). The addition of the dorsal burn injury not only augmented NF-κB activity in the injured muscle, but also prolonged it up to 14 days after injury (Figure 5, D and E), suggesting that burns provoke a systemic change that alters inflammatory signaling locally at sites of injury distant to the burn wound. Because the burned tissue becomes necrotic almost immediately after the injury, NF-κB activity was not significantly detected by bioluminescence at the wound in this model (Supplemental Figure 2).

### Inhibition of early plasmin activation reduces burn-induced systemic inflammation.

**Figure 6:**
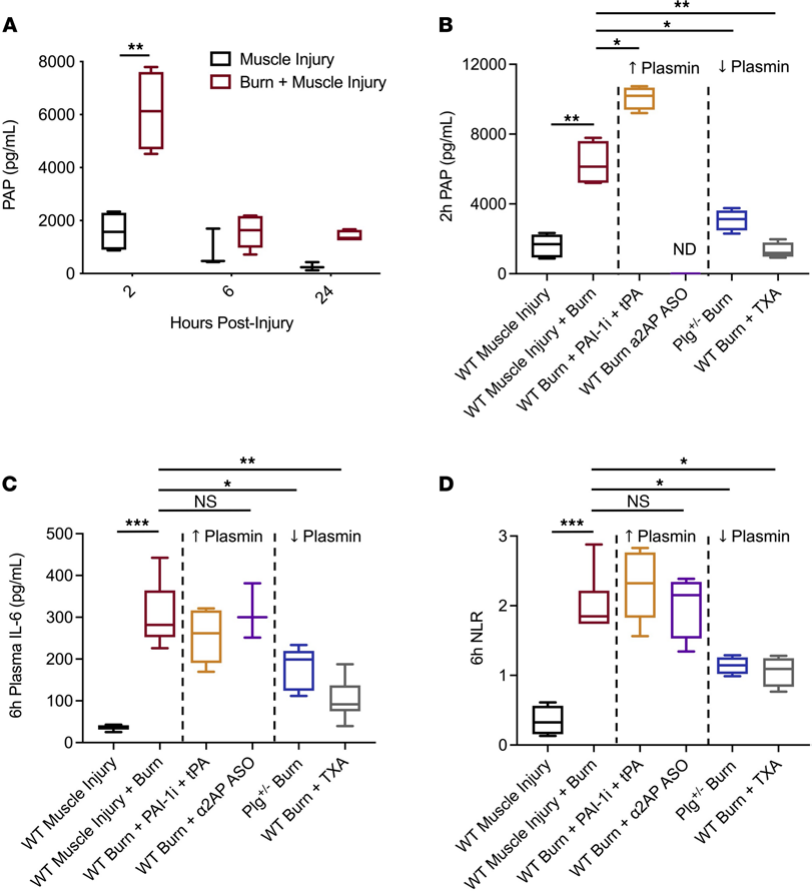
Inhibition of early plasmin activation reduces circulating inflammatory markers following burn. (**A**) Plasmin activity peaks within 2 hours of the injury and remains slightly elevated 24 hours following the burn injury. (**B**) PAI-1i + tPA was effective at increasing plasmin activation, while Plg^+/–^ mice and WT mice treated with TXA had reduced plasmin activation. As a part of the PAP complex, α2AP ASO resulted in PAP values below the limit of detection. ND, not detected. (**C** and **D**) Treatments to enhance plasmin activity (PAI-1i + tPA or α2AP ASO) did not alter inflammatory markers in the blood following burn, while Plg^+/–^ mice or WT mice treated with TXA exhibited reduced circulating markers of inflammation (IL-6 and neutrophil/lymphocyte-ratio [NLR]). Kruskal-Wallis with Dunn’s post hoc correction. *P < 0.05, **P < 0.01, ***P < 0.001. *n =* 4–5/group. Boxes represent median, while whiskers represent the 5th–95th percentile.

To determine the roles of plasmin in burn-induced systemic inflammation, we used pharmacologic and genetic tools to manipulate plasmin activity following burn injury in mice. Low-level PAP, plasma IL-6, and NLR were detected following an isolated muscle injury only (i.e., nonsevere APR). However, when the muscle was injured in conjunction with a burn (severe APR), significant plasmin activation (measured by PAP) was observed in the plasma of mice at 2 hours after injury, with resolution by 6 hours after injury (Figure 6A), aligning with clinical observation from burn patients (Figure 2B), although with different timing.

To assess the role of plasmin activity in burn-induced systemic inflammation, we enhanced plasmin activity or plasmin activation prior to causing a burn injury in WT animals. This was achieved through the administration of an inhibitor of plasminogen activator inhibitor-1 (PAI-1) in combination with an i.v. bolus of tissue plasminogen activator (tPA) at the time of injury or a validated antisense oligonucleotide (ASO) against plasmin’s primary inhibitor, α2-antiplasmin (α2AP) (48). PAP was not detectable in mice treated with α2AP ASO, as expected. However, an increase in PAP was observed in mice treated with a PAI-1 inhibitor in combination with tPA (Figure 6B). Regardless of the pharmacologic mechanism employed to enhance plasmin activity, no significant changes in plasma levels of IL-6 or NLR were observed at 6 hours after injury (Figure 6, C and D). To reduce early plasmin activation following injury, a burn injury was applied to mice with a 50% Plg deficiency (Plg^+/–^) or WT mice treated with clinical antifibrinolytic tranexamic acid (TXA) immediately following the burn. In mice with either genetic or pharmacologic reduction of plasmin or Plg, we observed a significant reduction in PAP at 2 hours after burn injury (Figure 6B). Furthermore, plasma levels of IL-6 and NLR at 6 hours after injury were significantly reduced by up to 50% compared with nontreated burn injuries (Figure 6, C and D). No significant differences were observed between groups at 24 hours (Supplemental Figure 3). There were also no differences observed between these groups in animals that received a muscle injury alone (not shown). Together, these data suggest that plasmin activity exacerbates systemic inflammation following a severe burn but that its role in burn-induced inflammation is maximized by the magnitude of the injury rather than the amount of plasmin activity.

### Inhibition of plasmin reduces the magnitude of NF-κB signaling in a remote muscle injury following burn.

**Figure 7:**
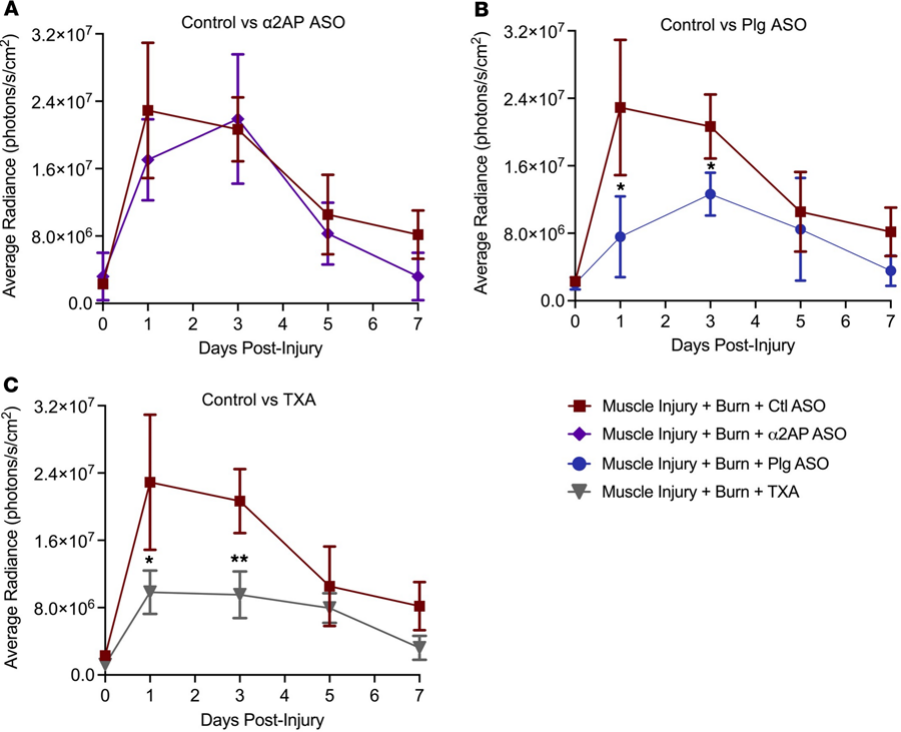
Inhibition of early plasmin activation following burn reduces NF-κB signaling in injured muscle. (**A**) Enhanced plasmin activity by α2AP ASO had no significant effect on muscle NF-κB activity. (**B** and **C**) Inhibition of plasmin activity by (**B**) Plg ASO or (**C**) TXA reduced NF-κB signaling in injured muscle following burn. No differences were observed across groups with muscle injuries alone (not shown). Repeated-measures ANOVA. *P < 0.05, **P < 0.01. *n =* 5/group; data points represent mean ± SD with 95% CI.

To determine if plasmin plays a role in tissue inflammation at sites of injury remote to the burn, mice expressing an NF-κB–inducible luciferase–GFP reporter gene (NGL) were challenged with either a muscle injury alone or a muscle injury with a burn and treated with α2AP ASO, TXA, or a validated plasminogen ASO (Plg ASO) to transiently knock down Plg expression (48). Enhancement of plasmin activity by α2AP ASO did not alter the magnitude of NF-κB activity in injured muscle (Figure 7A). While burned animals treated with TXA or Plg ASO still exhibited augmentation of NF-κB activity within injured muscle compared with the muscle injury alone, both methods of early plasmin inhibition significantly reduced the magnitude of the muscle NF-κB activity (Figure 7, B and C). These data suggest that plasmin activity following burn injury also exacerbates local inflammation within tissue at sites of injury remote to the burn, but this effect could not be exacerbated further with pharmacologic enhancement of plasmin activity in this model.

## Discussion

Previous studies suggest that burn injury does not provoke significant plasmin activation except in extreme cases of coagulopathy (39). However, we demonstrated significant burn-induced plasmin activation and fibrinolysis based on markers of in vivo plasmin activation and fibrinolysis. The clinical significance of severe injury–induced plasmin activation in the absence of bleeding was previously unclear. In this study, we observed a clinical association between markers of immunocoagulopathy and plasmin activation following burn injuries. To investigate a causative relationship between burn-induced plasmin activity and inflammation specifically, we implemented a mouse model of polytrauma that includes a severe burn injury with a remote muscle injury. The results of this study suggest that SIRS, measured by both circulating cytokines and distant tissue inflammatory signaling, occurs following burn and that the magnitude of this inflammatory response is, in part, mediated by plasmin; they also suggest that pharmacologically blocking early plasmin activity following burn attenuates SIRS — a key driver of thrombosis and MODS in injured patients.

Burn-induced SIRS has been shown to affect other tissues, and the consequences of severe burn–provoked inflammation on tissue health have been extensively demonstrated by the phenomenon of burn-induced osteoporosis and muscle wasting (49–51). Although we used a remote muscle injury to investigate how the systemic response to burn affects inflammatory signaling in distant tissues, tissue inflammation is often observed in a multitude of other organs following burn injuries, resulting in MODS (8, 52). Patients with severe injuries often present with polytraumas. These combinatory injuries, such as blast injuries, are frequently seen in military populations, as well as motor vehicle accident victims (44, 53). Furthermore, the majority of patients with severe burns undergo early surgical intervention, including excision and grafting, which introduces further tissue damage (54). Therefore, severe injury–induced inflammation is a critical target to reduce its pathologic effects on peripheral healthy or injured tissues. Future studies are required to determine if the effect of plasmin on tissue inflammation following burn is due systemic changes in circulating inflammatory mediators or if plasmin acts specifically at the site of the injury to augment inflammatory signaling.

Plasmin has a wide range of effects on inflammatory signaling through multiple mechanisms and interacts with many receptors on the surface of macrophages, neutrophils, and DCs to regulate cellular migration, phagocytosis, NETosis, and inflammatory signaling (15, 55). Following the early APR, plasmin has been shown to resolve inflammation by removing fibrin, inducing neutrophil apoptosis, and reprogramming macrophages within the injured tissues to antiinflammatory phenotypes (1, 55–57). Alternatively, plasmin has also been shown to activate inflammation through matrix metalloproteinase (MMP) activation (22, 58, 59), complement activation and bradykinin pathway activation (16, 35), macrophage chemokine release and TH17 cell activation (60), protease-activated receptor (PAR) signaling (15, 61), and NF-κB-mediated cytokine activation, and chemokine production in neutrophils and macrophages (24, 58, 62–65) in a time- and concentration-dependent manner. Plasmin can also exert proinflammatory effects through fibrinolysis; fibrin degradation products have been shown to increase release of inflammatory cytokines from monocytes (62, 66). Each of these pathways has been shown to be acutely activated at the site of significant injury, and while beneficial during tissue repair, these mechanisms have the potential to exacerbate inflammation during the early severe-APR, driving SIRS. Studies in severe injuries have shown that inhibition of plasmin to reduce bleeding has the off-target effect of reduced inflammation (21), and here we have shown that, independently of bleeding, early inhibition of plasmin following severe injury reduces markers of SIRS.

Our study serves to reveal the complexities of Plg activation and fibrinolysis in trauma and sheds light on the bases of the differences in fibrinolytic phenotypes observed across trauma patients (67, 68). A key observation highlighted by our studies is the acquisition of a hypoplasminogenemic state in the plasma of burn patients, but a major unanswered question here is the fate of the Plg. Loss of detectable Plg could be due to both redistribution and activation-mediated consumption. Each of these possibilities could be linked to the immense volume of fibrin deposited within the zone of coagulation of a burn. The damaged tissue milieu, including fibrin, presents multiple binding sites for Plg in both the intra- and extravascular spaces, and these provide an evolutionary advantage of localizing plasmin activation to the site of coagulation or tissue injury. However, in polytraumas, this could cause circulating Plg to preferentially bind to larger areas of tissue damage, while smaller injuries requiring plasmin for repair, such as muscle, may become transiently deficient in plasmin. Furthermore, injury-induced, blood hypoplasminogenemia has the potential to affect some diagnostic assays measuring fibrinolytic potential in the blood and may falsely indicate a hypofibrinolytic condition, even though in vivo markers of plasmin activation and D-dimer indicate differently. Currently, the clinical significance of this Plg redistribution is unknown, but in this study, the loss of circulating Plg retrievable by blood draw also correlated with the degree of organ failure incurred in burn patients within the first week of hospitalization, and this may reflect the magnitude of tissue damage.

In burn, plasmin activation may be missed due to timing of admission. PAP levels were only slightly elevated in later samples after burn, regardless of burn severity, suggesting that plasmin activation is a rapid and transient occurrence that may be poorly detected using measurements such as TEG. Because burns represent a large surface area of tissue damage with little to no risk of bleeding, in contrast to a penetrating trauma, it is possible that pathologic plasmin activation is occurring more locally without a systemic release of Plg activators into the blood that would result in significant clot lysis by TEG measurements. Although TEG is clinically useful to predict bleeding and inform resuscitation in certain types of injury and disease (39, 69, 70), in order to detect plasmin activation in different types of severe injury without bleeding risk, other markers of plasmin activation, such as D-dimer or PAP, may be required in addition to TEG.

While this study aimed to provide a comprehensive translational study, there were limitations. The human burn patients analyzed had variable burn severities in order to measure the heterogeneous responses to the injuries; however, some of these patients had minor, secondary injuries and differing surgical interventions along with the burns that may have added variability to the cohort. Alternatively, while the mouse burn-polytrauma model was consistent, it did not reach the severity of some of the human burns and, therefore, did not provoke as severe of a systemic response or death. Future studies are needed to evaluate the benefits of therapeutically targeting plasmin to reduce inflammation and organ dysfunction in larger animal models with greater injury severity. Furthermore, larger animal models with comparable severity may be used in the future to investigate the timing of the APR and extrapolate these findings to human trauma patients to overcome variable timing of admission. Ideally, future clinical and preclinical studies in severe injuries would incorporate standard clinical laboratory tests with earlier markers of the APR, such as IL-6, plasmin activation, and Plg levels, to assess the magnitude and timing of the APR, even in the absence of bleeding, in order to inform appropriate resuscitation and surgical intervention and to predict the risk of adverse outcomes.

The inflammatory response to injury is an crucial, complex sequence of events, and based on a collective of studies, plasmin does appear to play a paradoxical role in inflammation following injury, depending on timing and location (56, 58). Human studies in trauma have shown a time-dependent effect of TXA on patient outcomes: early administration of antifibrinolytics reduces blood loss and inflammation, but later administration increases the risk of bleeding and/or thrombosis (19, 71, 72). While plasmin has been shown to exacerbate an initial inflammation in infection and injury, it has conversely been shown to be equally important in resolving the inflammatory response (24, 73), making plasmin essential later for recovery and for the repair of all tissues (1, 74–76). In this study, while early inhibition of plasmin activity reduces markers of inflammation, enhancing plasmin activity early in convalescence did not increase inflammatory markers, suggesting that plasmin activity may be maximized in this model or that plasmin’s proinflammatory effects may be dependent on the magnitude of injury. Alternatively, enhancing plasmin activity later following a severe injury may promote repair, while inhibiting plasmin later may have pathologic effects on tissue repair; however, this requires further study. Collectively, our study highlights that the potential therapeutic benefit to inhibiting plasmin following trauma is time dependent, and therefore, accurate in vivo diagnostic measures of plasmin activity are required to inform antifibrinolytic therapy.

Trauma-related deaths have been observed to occur in different peaks over time: immediate, early, and late deaths (77). Recent research has demonstrated that medical advances have greatly reduced the late deaths, but those within the first day of injury due to hemorrhage, brain injury, and early organ dysfunction remain unchanged (25, 26). Approximately 30%–40% of deaths occur within several hours of the injury, and 10%–20% of deaths occur beyond 24 hours after injury. The early peak of mortality is primarily attributed to hemorrhage and brain injury, and the later deaths are attributed to MODS and infection (26, 77). In burn specifically, deaths occur later, as there is little risk of early hemorrhage; however, the majority of burn-related deaths within the first week occur due to SIRS-associated MODS or sepsis (78, 79). This study and others have suggested that timely inhibition of plasmin may reduce injury-associated SIRS, and interestingly, recent studies in surgery have demonstrated that inhibition of plasmin reduces injury-associated immunosuppression and subsequent risk of postsurgical infection (80, 81). Plasmin’s role in infection prevention should be studied in other injury models in the future. While antifibrinolytics are often used to reduce the risk of bleeding-associated mortality within hours of the injury (82), the findings of this study suggest that antifibrinolytics may be used to reduce the risk of mortality during the late peak due to an immunocoagulopathic condition observed following the injury.

### Conclusion.

Severe burn injuries provoke clinically significant plasmin activation. Although hyperfibrinolysis and subsequent bleeding have been the areas of primary concern for plasmin activation following severe injury, plasmin also plays a role in severe injury–induced SIRS in the absence of bleeding. The results of this study suggest that early inhibition of plasmin following severe injury by antifibrinolytics may provide further therapeutic benefits in patients with severe injuries by reducing SIRS and the risk of SIRS-related complications.

## Methods

### Human burn patients.

Burn patients admitted to the Vanderbilt University Medical Center burn unit with ≥ 5% TBSA burns were prospectively enrolled into this study upon consent from either the patient or closest family member. Patients with electrical burns, multiple severe injuries, or Stevens-Johnson syndrome were not included in this study. 31 burn patients were recruited in total. Small blood samples were collected from burn patients at 0.5, 1, 3, 5, and 7 days after burn or until discharge or mortality. Three patients requested cessation of blood sampling at 5 days after admission. Demographic data were collected from patient charts including age, sex, and BMI. Study-related data collected from the charts included TBSA affected, the presence of inhalation injury, routine complete blood counts throughout hospitalization, relevant surgical procedures, and mechanism of burn. Burn severity was calculated using the revised Baux score as previously described (83) to account for patient age, TBSA, and the presence of inhalation injury. Serial creatinine, bilirubin, FiO_2_, PaO_2_, platelet counts, and blood pressure values taken from routine laboratory tests and Glasgow Coma Scale were collected for calculation of SOFA as previously described (27). A single blood sample was collected from 15 healthy individuals to serve as controls.

### Animals.

For all experiments, 6- to 8-week-old, 18–21 g male and female mice on a C57BL/6J background were used with age- and sex-matched animals for each treatment and control group. WT mice were obtained from the Jackson Laboratory, and Plg-deficient mice background were obtained originally from the lab of Jay Degen (Cincinnati Children’s Hospital, Cincinnati, Ohio, USA). For in vivo quantification of NF-κB activity, transgenic mice expressing GFP and luciferase downstream of an NF-κB–activated 5′ HIV-LTR promoter (NGL) were used as previously described (47). WT and NGL mice were provided standard laboratory chow, and Plg-deficient mice and comparable WT littermate controls were provided synthetic laboratory chow (Research Diets Inc.) and water ad libitum. All mice were housed in a 12-hour light-dark cycle within a designated animal facility at Vanderbilt University.

### Burn injury model.

To assess the systemic effects of severe burn on local and systemic inflammation, a well-established model of burn injury (43) was employed. Thirty minutes prior to the burn, mice were administered 0.1 mg/kg s.c. buprenorphine. Under 3.0% isoflurane general anesthesia, mice were shaved along the dorsum, and 1 mL of sterile saline was injected s.c. along the spine to prevent injury to deep tissues during the burn. Bilateral calf muscle injuries were administered by i.m. injections of 10 μM cardiotoxin, as previously described (45). Afterward, the mice were placed in a heat-resistant template with a cutout to expose the dorsum and submerged in 100°C water bath for 10 seconds to create an approximately 30% TBSA, full-thickness burn to the dorsum. Immediately following the burn, a resuscitative i.p. injection of 2 mL Lactated Ringer’s solution was administered, and mice were monitored for 30 minutes following the injury. A maintenance dose of buprenorphine was administered every 8–12 hours for 72 hours, and all animals were housed in individual sterile cages following the burn injury. Experimental controls received the same treatment and muscle injuries in the absence of burn. Blood was collected at 2, 6, 24, and 48 hours after burn for serological analysis.

### Pharmacologic manipulation of plasmin activity.

To inhibit plasmin activation, clinical antifibrinolytic drug TXA was administered at 1000 mg/kg i.p. beginning immediately after the burn and every 3 hours up to 9 hours after burn, and control animals received injections of saline. Alternatively, mice received either a validated ASO against Plg (IONIS Pharmaceuticals) to knock down Plg expression or a nontargeted control ASO (48). All ASOs were developed and provided by IONIS Pharmaceuticals. Plg (5′ AGTGATGGTCTATTGTCACA 3′) and control ASOs (5′ CCTTCCCTGAAGGTTCCTCC 3′) were administered at 330 mg/kg by s.c. injection weekly beginning 2 weeks prior to injury and continuing for the duration of the study. These ASOs were synthesized, purified, and tested for efficacy as previously described (48).

To enhance existing plasmin activity provoked by the burn, animals received a validated ASO against plasmin’s primary inhibitor, α2AP (5′ CACTGGTGATGGTCCTTCCG 3′) or control ASO as described above. To increase the amount of plasmin activated at the time of injury, a combination approach was implemented to significantly increase plasmin activation at the time of burn. Mice were placed on synthetic laboratory chow supplemented with a specific inhibitor of PAI-1, MDI-2517 (MDI Therapeutics Inc.) received from Daniel Lawrence at University of Michigan (Ann Arbor, Michigan, USA). The PAI-1 inhibitor was administered at 500 mg/kg of food beginning 1 week prior to the procedure, and the mice also received an i.v. injection of 10 mg/kg recombinant human tPA (Activase, Genentech) as previously described (84) at the time of burn. Control animals received synthetic chow without PAI-1 inhibitor 1 week prior to the procedure and were administered i.v. saline at the time of burn.

### In vivo imaging.

Imaging of injured muscle for bioluminescence was conducted using the Xenogen IVIS Spectrum (PerkinElmer). Under brief anesthesia, NGL mice received a retro-orbital injection of 75 mg/kg D-luciferin (PerkinElmer) in sterile saline. Mice were imaged within 5 minutes of injection with an exposure time of 1.0 second. Image quantification and analysis were conducted using IVIS Living Image 4.3.1 software (PerkinElmer). All mice were imaged prior to injury and 1, 3, 5, and 7 days after injury, and a subset of mice were imaged at 10 and 14 days after injury.

### Blood and plasma measurements.

For human plasma measurements, citrated blood was centrifuged at 1500*g* and then 13,000*g* for 15 minutes each to produce platelet poor plasma (PPP). Plasma samples were analyzed based on availability. Commercially available and validated ELISAs were used to measure plasma S100A10 (p11, Abbexa), Plg, and PAP complexes (Molecular Innovations). Plasma P-selectin, CD40L, IL-6, IL-1β, C5a, uPA, suPAR, L-selectin, and D-dimer were measured on custom multiplex panels using validated, commercially available antibodies at a 1:2 plasma dilution (R&D Systems). Plasma Plg levels were measured using a fluorogenic plasmin activity assay (adapted from Abcam, ab204728). Plasma samples and control pooled plasma (George King Bio-Medical Inc.) were diluted 1:10, 1:20, and 1:50 in HEPES buffer. Diluted plasma and fluorogenic substrate (420 μM final) (H-D-Val-Leu-Lys-AFC, Anaspec) were added in duplicate to the wells of a 96-well plate. Plasmin activation was initiated by the addition of 0.5 U/μL streptokinase (MilliporeSigma), and fluorescence (excitation at 380 nm, emission at 500 nm) was measured every 30 seconds for 1 hour using a Synergy 2 plate reader (Biotek). Initial rates of plasmin generation were calculated, and Plg levels were reported as a percent of pooled control plasma.

For mouse plasma measurements, PPP was isolated as described above, and IL-6 (1:2 plasma dilution) (Abcam) and PAP (1:100 plasma dilution) (Biotang USA) were measured by validated ELISA. Mouse complete blood counts were measured using the Forcyte Hematology Analyzer (Oxford Science) at the Vanderbilt University Medical Center Translational Pathology Shared Resource.

### Statistics.

Power sample calculations were conducted using PS version 3.1.6 for both human and animal experiments. For humans, based on the difference of means, SD, and cohort ratio for the different measures, for α = 0.05 and power = 0.80, the minimum sample size for each group was 7. For animal experiments, in order to detect a difference between treatment groups with α = 0.05 and power = 0.80 and a ratio of 1:1 for all groups, the minimum sample size required was 4.

To compare groups at different time points with a control group in both humans and animals, Kruskal-Wallis tests were performed with post hoc Dunn’s test to correct for multiple comparisons. Pearson’s correlation was used to determine a linear associative relationship between variables, including plasmin activation, inflammation, coagulopathy, and patient outcomes. For Kaplan-Meier analysis, a log-rank test was used to determine predictors of mortality within 7 days of burn. To compare injury or treatment groups at multiple time points in animal experiments, multiple Mann-Whitney *U* tests were performed with Holm-Sidak correction for multiple comparisons between groups over time. For comparison of repeated measures over time, a repeated-measures ANOVA was performed. All tests were performed in GraphPad Prism version 9.1.2.

### Study approval.

All human study procedures were approved by the Vanderbilt University Medical Center IRB (protocol no. 150751). All animal study procedures were approved by the VUMC IACUC (protocol no. M1800154).

## Author contributions

BHYG was responsible for study design, execution of experiments, acquiring and analyzing data, and manuscript preparation. CCW, SNML, and PRK conducted experiments and assisted with sample collection. JBS assisted with study design, patient recruitment, and sample collection. ARR, TSB, and MJF provided key reagents and/or animals and assisted with study design. JGS contributed to study design, data analysis, manuscript preparation, and study funding. All authors provided critical revisions to the manuscript and have approved the final draft.

## Supplementary Material

Supplemental data

## Figures and Tables

**Table 1:**
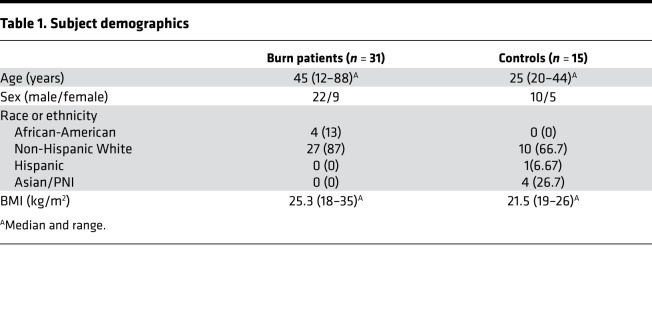
Subject demographics

**Table 2:**
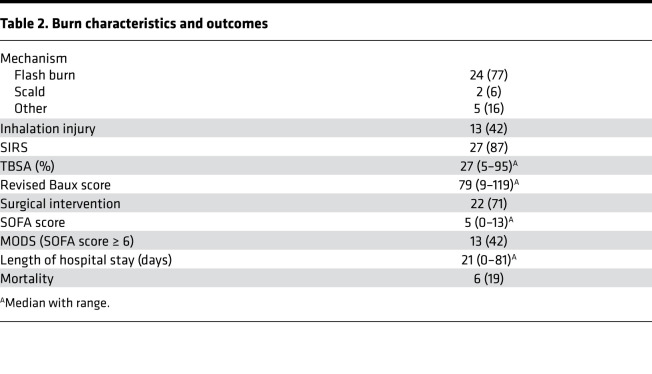
Burn characteristics and outcomes

## Version 1 12/08/2021

Electronic publication
