# Supplementary material for: Plasmin drives burn-induced systemic inflammatory response syndrome
Source: JCI Insight. 2021 Dec 8;6(23):e154439. doi: 10.1172/jci.insight.154439 (PMC8675186; doi:10.1172/jci.insight.154439)
Supplement: Supplemental data [file jciinsight-6-154439-s138.pdf]

SUPPLEMENTAL FIGURES

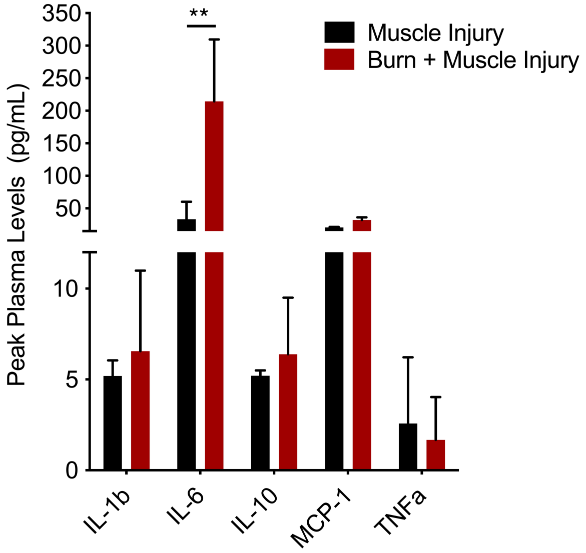

Figure S1: Of the cytokines measured, IL-6 exhibited the strongest response to a sterile, 30% TBSA burn injury with calf muscle injuries compared with muscle injuries alone.

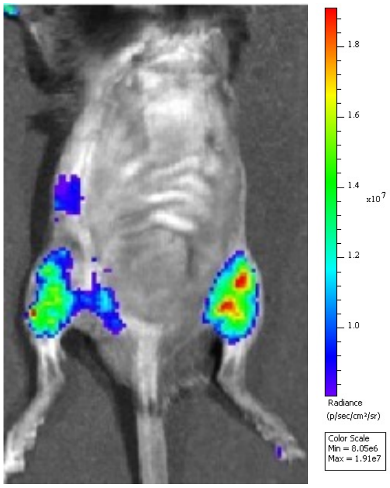

Figure S2: Bioluminescent signal in mouse with burn and bilateral calf muscle injuries.

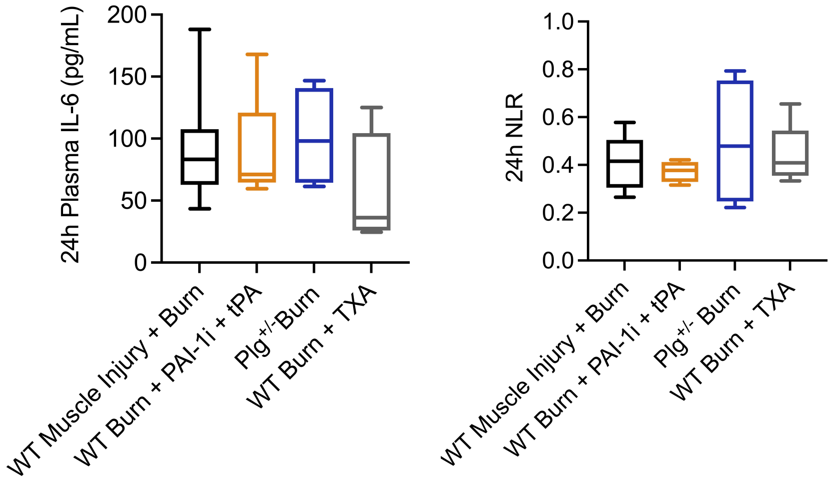

Figure S3: Enhancement or inhibition of plasmin did not significantly alter A) plasma IL-6 or B) NLR at 24h following the burn injury. (Kruskal-Wallis was performed)
